# Automating document classification with distant supervision to increase the efficiency of systematic reviews: A case study on identifying studies with HIV impacts on female sex workers

**DOI:** 10.1371/journal.pone.0270034

**Published:** 2022-06-30

**Authors:** Xiaoxiao Li, Amy Zhang, Rabah Al-Zaidy, Amrita Rao, Stefan Baral, Le Bao, C. Lee Giles

**Affiliations:** 1 Department of Statistics, Pennsylvania State University, University Park, PA, United States of America; 2 Information and Computer Science Department, King Fahad University of Petroleum and Minerals, Dhahran, Saudi Arabia; 3 Department of Epidemiology, Johns Hopkins University, Baltimore, MD, United States of America; 4 College of Information Sciences and Technology, Pennsylvania State University, University Park, PA, United States of America; Danube Private University, AUSTRIA

## Abstract

There remains a limited understanding of the HIV prevention and treatment needs among female sex workers in many parts of the world. Systematic reviews of existing literature can help fill this gap; however, well-done systematic reviews are time-demanding and labor-intensive. Here, we propose an automatic document classification approach to a systematic review to significantly reduce the effort in reviewing documents and optimizing empiric decision making. We first describe a manual document classification procedure that is used to curate a pertinent training dataset and then propose three classifiers: a keyword-guided method, a cluster analysis-based method, and a random forest approach that utilizes a large set of feature tokens. This approach is used to identify documents studying female sex workers that contain content relevant to either HIV or experienced violence. We compare the performance of the three classifiers by cross-validation in terms of area under the curve of the receiver operating characteristic and precision and recall plot, and found random forest approach reduces the amount of manual reading for our example by 80%; in sensitivity analysis, we found that even trained with only 10% of data, the classifier can still avoid reading 75% of future documents (68% of total) while retaining 80% of relevant documents. In sum, the automated procedure of document classification presented here could improve both the precision and efficiency of systematic reviews and facilitate live reviews, where reviews are updated regularly. We expect to obtain a reasonable classifier by taking 20% of retrieved documents as training samples. The proposed classifier could also be used for more meaningfully assembling literature in other research areas and for rapid documents screening with a tight schedule, such as COVID-related work during the crisis.

## Introduction

We are at a pivotal time in the global HIV response as progress towards ending the HIV pandemic by 2030 is off-track. A major reason is that not all have benefited from the advances in treatment and pre-exposure prophylaxis (PrEP) [1, 2]. An estimated 8% of new adult infections globally were among sex workers who are at 30 times greater risk of acquiring HIV than other reproductive-aged people [3]. Unfortunately, HIV-related data among female sex workers are still limited due to sustained individual and structural stigmas, including criminalization [4]. In both the Global Fund 2017–2021 strategy and President’s Emergency Plan for AIDS Relief (PEPFAR) 3.0, the need for empirical data-driven responses was highlighted as central to informing an effective HIV response. As such, we are building data repositories for particular communities disproportionately affected by HIV, generally called key populations.

A comprehensive database that collects existing findings of sex workers can be leveraged to develop better strategies for optimizing the impact of HIV prevention and treatment programs [5]. Such a database could be created through a systematic literature review (SR), a key step of evidence-based public health programs, which distinguishes itself from ad hoc literature reviews and selection by its explicit and systematic approach [6]. Studies for specific diseases or interventions are collected, summarized, and extensively reported via SR to aid empiric decision-making by physicians, policymakers, and patients. Examples include the global burden of disease attributable to mental and substance use disorders [7], the long-term health consequences of child abuse [8], the effect of antibiotic prescribing on antimicrobial resistance [9], and so on. As such, guidelines for conducting and reporting SRs have been developed to improve the quality of SRs and increase their transparency. One notable example is the Preferred Reporting Items for Systematic Reviews and Meta-Analyses (PRISMA), which consists of a 27-item checklist and four-phase diagram to help improve the reporting of systematic reviews [10]. There are five steps in forming an SR study [11]: (1) Formulating the research questions; (2) Identifying relevant work through an exhaustive search of the literature; (3) Assessing the quality of studies using; (4) Summarizing the evidence through tabulating study characteristics, quality, and effects as well as statistical methods for meta-analysis; (5) Interpreting the findings. Among those, Step (2) is particularly time-demanding and labor-intensive. To complete high-quality systematic reviews, one must invest significant efforts into developing and implementing appropriate search strategies, including searching through tens of thousands of research articles to include in an SR.

Here, we provide automatic document classification (ADC) procedures to reduce the manual labor involved in the systematic review process. We use a SR of identifying studies with HIV impacts on female sex workers as a case study, and this study represents the first attempt of automating the HIV and key population systematic reviews.

We first systematically reviewed all published literature related to female sex workers under the PRISMA guidelines, and then assembled data from studies published in all low and middle-income countries, including all Sub-Saharan African countries except for Seychelles. To compare with a traditional systematic review, we used ADC to identify journal articles that characterize key features of female sex workers as our baseline.

Multiple document classification models were implemented and evaluated for their performance by different metrics and sensitivity analysis. Our proposed method for document classification refines the high-dimensional encoding associated with bag-of-words to the *N* most important words for classification purposes, reducing the dimension of the feature space and improving performance. We demonstrated this using the random forest algorithm because of its ability to handle a large number of noisy predictors, to allow for high-order interaction effects, and to prevent over-fitting. The feature screening and extraction explored in the paper can be generalized for different machine learning algorithms. We showed additional examples of Support Vector Machine (SVM), Boosting, Neural Network and ElasticNet, and compared their performance in our case study.

## Materials and methods

In this section, we first introduce the procedure for manually reviewing and labeling the status of articles. The results of manual document classification will be used as a gold standard and as a means to evaluate the performance of automated data classification methods. We then describe the natural language processing (NLP) tools for preprocessing the text data. The extracted tokens will serve as binary predictors for the automated classification algorithms. Finally, we propose a series of document classification models for automatically classifying documents into the relevant class versus the irrelevant class.

### Manual document classification and traditional systematic review methods

**Fig 1:**
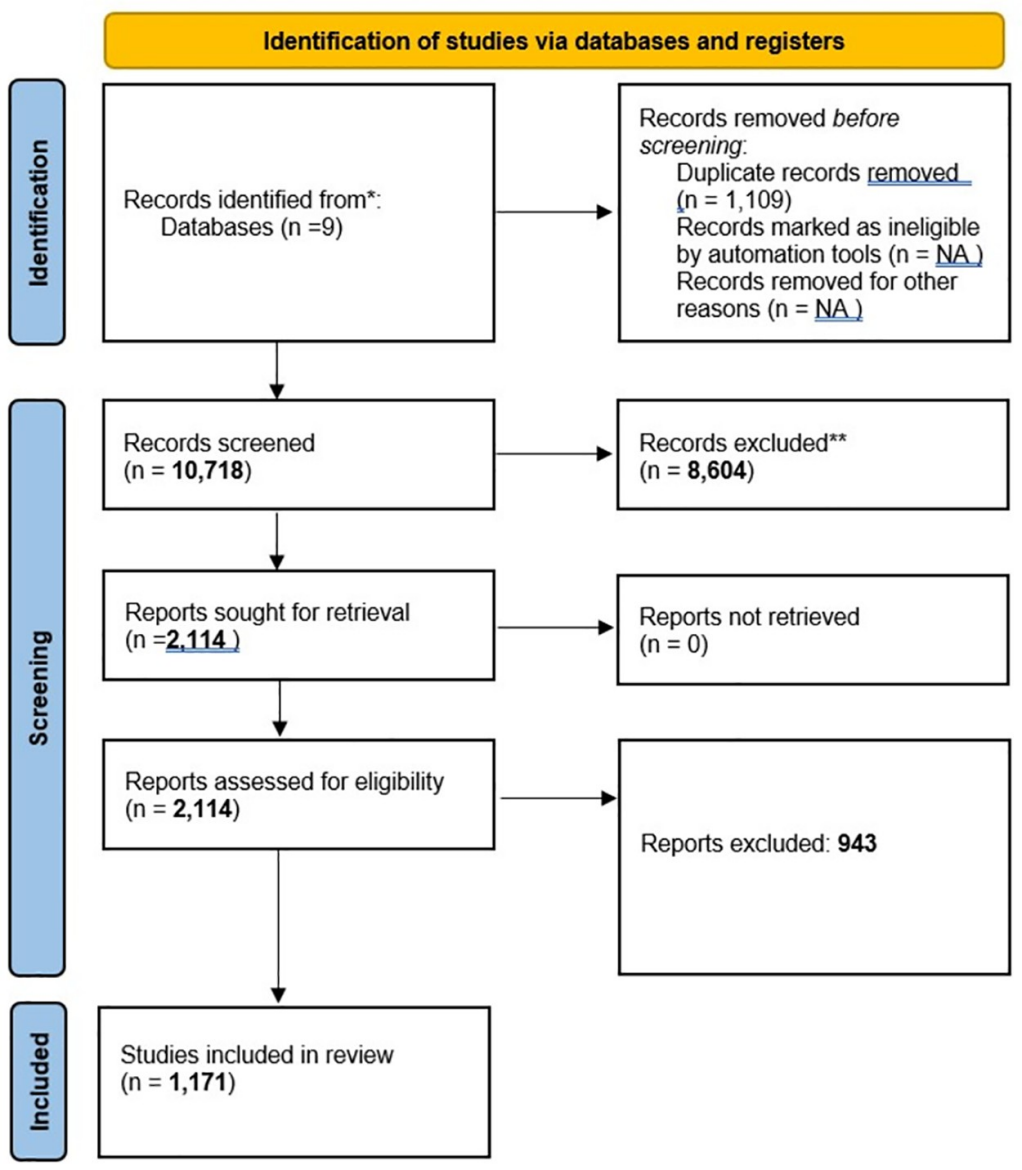
Flow diagram for systematic reviews of HIV prevalence, the HIV treatment cascade (HIV testing, linkage to care, treatment, viral suppression) and experienced violence (physical, sexual, and intimate partner) among among female sex workers.

We obtained 10, 718 journal articles which contained relevant keywords from PubMed, EMBASE, Global Health, SCOPUS, PsycINFO, Sociological Abstracts, CINAHL (Cumulative Index to Nursing and Allied Health Literature), Web of Science, and POPLine. The search strategy and keywords used are detailed in the keyword-guided document classification model. The identified articles were labeled as relevant if they included HIV prevalence, the HIV treatment cascade (HIV testing, linkage to care, treatment, viral suppression), or experienced violence (physical, sexual, and intimate partner) among female sex workers and labeled as irrelevant otherwise. The labeling process consisted of an initial screening stage and a final selection stage. In the screening stage, a team of reviewers, two independent reviewers per article, using standardized processes reviewed titles, abstracts, and citation information to determine the article’s relevance. The full text of articles marked as potentially relevant was then extracted and reviewed by the same team of reviewers, again using two independent reviewers per article, using a standardized approach to determine the final selection. Differences were resolved through consensus and referral to a senior study team member when necessary. Both the initial screening and final reviews were conducted using Covidence, a tool designed to help facilitate the systematic review process. The systematic review protocol has been published elsewhere [12] and is registered in the PROSPERO database (CRD42016047259; 28 September 2016). The flow diagram describing the screening and the review process and which follows PRISMA guidelines is presented in Fig 1.

In addition to subject matter constraints, all articles and reports used in the study must have been published in a peer-reviewed journal, presented as an abstract at a scientific conference, or available on the web from governmental or non-governmental sources between 2006 and 2017. Works published in languages other than English and studies where the sample size was less than 50 were not included. Some documents in the database had no abstract information and were also excluded during analysis.

### Data preprocessing for automatic document classification

Data preprocessing was conducted using the tidytext [13], quanteda [14], and tm [15] packages in R [16]. We combined the titles and abstracts of the documents and normalized the raw text data into tokens as follows:

**Tokenization**: We split the text into individual word tokens based on white space and segmented the text into basic linguistic units. All stop words, punctuation, numbers, special characters, and languages other than English were removed.

**Lemmatization**: Following Mechura’s [17] English lemmatization list, we grouped together various derivative forms of a word which share similar semantic meaning such that they could be analyzed as a single item.

**Stemming**: We removed word suffixes and conflated the resulting morphemes with the Porter stemmer [18] which leads to a crude affix chopping. For example, “automates” and “automation” all reduce to “automat” using the Porter stemmer.

The tokens obtained after pre-processing for each document were translated into a numerical vector representation called the document-term-matrix (DTM). A typical representation of the DTM is a *D* × *P* matrix, where *D* is the number of documents and *P* is the number of unique tokens after pre-processing. The (*d*, *i*)^*th*^ entry in the DTM records the frequency of token *i* in document *d*. Note that many tokens are common across documents but may not be relevant to the SR search criteria, such as articles, prepositions, and certain common verbs (e.g., “give”, “perform”). Thus we scaled token frequency within a document by the inverse of a token’s frequency across all documents, which is called term-frequency-inverse-document-frequency (TFIDF) [19]:

$$\begin{array}{c} {\text{tfidf}}_{i,d}={\text{tf}}_{i,d}\cdot {\text{idf}}_{i}, \end{array}$$ (1)

where tf_*i*,*d*_ is the term-frequency for token *i* in document *d* and idf_*i*_ is the inverse document frequency for token *i*. For our study, we used the logarithmically scaled inverse fraction of documents containing token *i*: ${\text{idf}}_{i}=\text{log}\frac{N}{\text{df}(i)}$ where *N* is the total number of documents and df(*i*) is the frequency of token *i* in documents. Using TF-IDF, tokens which are common across all documents are treated as less informative and thus less important. This measures how much information the word provides.

### Document classification models

We compared three classifiers that automatically identify articles of interest based on query searching, clustering, and a broadly used machine learning algorithm. The first model is a keyword-guided filtering approach and serves as the baseline model. The second model refines the tokens extracted from the abstracts of the documents into related clusters and uses the clusters as features in a random forest (RF) model, which we refer to as “RF with refined clusters”. The third model includes both the refined clusters and additional tokens screened for relevance in the random forest model, which we refer to as “RF with top tokens”. These are described in detail in the following subsections.

#### Keyword-Guided approach

Our goal was to identify documents with studies of female sex workers, which included HIV or violence data. As a baseline model, we only examined document classification based on a keyword-guided filtering approach. We retrieved articles based on both their Medical Subject Headings (MeSH) and the occurrence of any related terms within the body of the article, referred to as a Text Word (TW) query. MeSH is a specific dictionary of keywords maintained by the National Library of Medicine to index and catalog biomedical information. A MeSH query retrieves articles that the author has listed as belonging to that category. We identified keywords across three relevant categories: female sex workers (FSW), human immunodeficiency virus (HIV), and violence (Violence). The three categories were combined in a query with the structure “FSW AND (HIV OR Violence)” under a Boolean search algorithm [20]. Table 1 lists MeSH and TW key words for each category.

**Table 1:** Medical Subject Headings (MeSH) and Text Words (TW) used to retrieve relevant articles.

Category	MeSH	TW
Female Sex Workers (FSW)	Prostitution, Sex Worker	prostitut*, commercial sex, transactional sex, sw, fsw, csw, sex trade, trade sex
HIV	HIV, acquired Immunodeficiency Syndrom, HIV Infections	human immunodeficiency virus*, acquired immunodeficiency syndrome*, HIV*, AIDS
Violence	Violence, Domestic Violence, Workplace Violence, Crime Victims, Battered Women, Rape, Homicide, Coercion	Violen*, crime*, offense*, abuse*, victim*, rape*, assault*, batter*, extort*, intimidat*, exploit*, IPV, IPSV

* Represents usage of a wildcard operator in the database query, e.g.“violen*” returns both “violence” and “violent”.

#### Cluster refinement

The baseline keyword-guided model can be viewed as a tree-based classifier with three major predefined clusters of terms. We further divided those three large categories into finer clusters based on word roots, and allowed more flexible structures for classification. Constructing the finer clusters from the terms we extracted was a nontrivial process. Popular stemming methods truncate the ends of words, which often includes the removal of derivational affixes. Although the stemming process performs well in terms of reducing dimensionality, it also has the potential of creating ambiguity [21]. Due to the “crude chopping” of the stemming procedure, many outputs are not recognizable words. For example, “stay” becomes “stai” using the Porter stemmer. Also, affixes are essential in meaning in English, but stemmers fail to capture this effect extensively; for example, “recondition” shares a stem but not the root meaning of “recondite.” These issues may result in inaccurate stemming of words that share the same root but have different meanings, such as “absolutely” and “absolution.” To mitigate this problem, we grouped words of similar semantic meaning to guarantee that the same root can represent major words in the three categories. As a result, we partitioned the three major categories of tokens into the following 15 “finer” clusters: “hiv”, “fsw”, “violence”, “offense”, “abuse”, “torture”, “rape”, “victim”, “assault”, “harass”, “extort”, “homicide”, “coercion”, “ipv”, “exploit”.

To obtain the vector representation of those 15 clusters, we created a document-cluster-matrix and calculated TF-IDF based on the combined frequencies of the terms in each cluster. Each document was represented by a 15-dimension vector of TF-IDFs. We then used random forest [22] to classify the documents with 15 features. Random forest uses a bagging ensemble method and decision trees constructed by a subset of data to provide more stable and accurate classification results.

The two classes of documents were highly imbalanced: around one relevant document for every ten irrelevant ones. To relieve the distortion, we randomly down-sampled observations from the irrelevant class and kept similar sizes between the two classes when training each branch of the classification tree. We generated 500 classification trees, counted the votes for document *d* being relevant, *Y*_*d*_ = 1, among the 500 constructed trees, and used the proportion of votes as the fitted probability of $\hat{P}\left(Y_{d}=1\right)$. The R Package we use for implementing the random forest algorithm is **RandomForest** [23].

#### Top N tokens model

In addition to the three major categories for tokens used in the baseline and cluster-refinement models, there still may have been some tokens that contained important information for classification purposes. We introduced a feature screening method to identify other significant tokens and included these tokens as additional covariates in the classification model. For each token *i*, we used a two-sample t-test to determine whether its mean TF-IDF differed statistically between the relevant class and the irrelevant class. The test statistic was ${\bar{x}}_{i1}-{\bar{x}}_{i2}$ divided by the un-pooled variance, where ${\bar{x}}_{i1}$ is the mean TF-IDF value for token *i* within the relevant documents and ${\bar{x}}_{i2}$ the mean TF-IDF value for token *i* within the irrelevant documents. If a token *i* was useful for distinguishing between the two groups/classes, the t-statistic was expected to have a larger absolute value. We ranked tokens based on their absolute t-statistics and picked the top 20, 50, 100, 250, and 500 tokens as new features in addition to the 15 clusters defined in the cluster-refinement model and constructed a set of models using these top N features.

In addition to RF with refined clusters and RF with top tokens, we also explored a few other classifiers that could handle a large number of predictors. They are Support Vector Machine (SVM) implemented in the R Package **e1071** [24], Boosting implemented in the R Package **ada** [25], Neural Network implemented in the R Package **RSNNS** (Stuttgart Neural Network Simulator) [26], and ElasticNet implemented in the R Package **glmnet** [27]. All of the R packages were implemented in R environment, version 4.0.3 [16].

We trained each model using 5-fold cross-validation and evaluated their performance using the manually-assigned labels, described in the Data section, as ground truth. We used the receiver operating characteristic (ROC) curve and precision and recall (PR) curve as evaluation methods. ROC curve plots the true positive rate against the false positive rate; PR curve plots the precision rate against recall rate. Both ROC and PR curve illustrate the diagnostic ability of a binary classifier with varying discrimination threshold. All models except the baseline model produce probabilistic classifications; thus, we could additionally compare ROC and PR curves by varying the cut-off probability for classifying document *d* as relevant. We also reported the area under the curve (AUC) as a summary metric for these models, i.e. the integration of the area under ROC curve or PR curve. We used the caret [28] package in R, which includes convenient methods for re-sampling, model training, parameter tuning, and cross-validation for a large variety of models.

## Results

Following the manual document classification procedure, we initially obtained 10, 718 documents through keyword search, and identified 1, 171 papers as relevant and 9, 547 as irrelevant. We then evaluated the performance of automatic document classification (ADC) procedures by taking the manual classification results as the gold standard.

**Fig 2:**
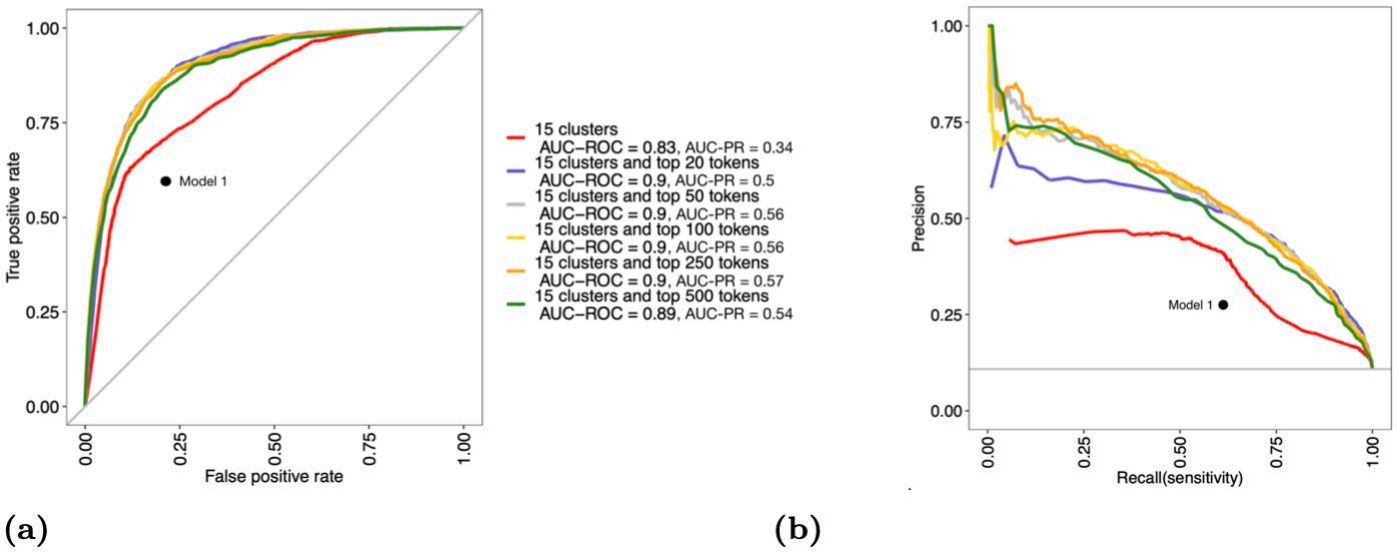
(a) ROC and (b) PR curves for the baseline model (keyword-guided approach), RF with 15 refined clusters and RF with top tokens (20, 50, 100, 250, and 500 tokens in addition to 15 clusters). As a summary metric, area under the curve (AUC) is provided in the legend.

Fig 2(a) shows the ROC curves of all models for comparison. RF with 15 refined clusters outperforms the keyword-guided approach of the baseline model as the ROC curve of RF (red) is well above the point that corresponds to the deterministic classification of the baseline model. Table 2 shows that RF with top tokens further improves the area under the ROC curve (AUC-ROC) from 0.83 to 0.90. Varying the number of additional tokens (from *N* = 20 to *N* = 500) in RF does not obviously change its performance.

**Table 2:** The area under the curve (AUC) for ROC and PR of random forest models. All models include 15 refined clusters. The additional number of tokens is shown as the column names.

Metric	0 tokens	20 tokens	50 tokens	100 tokens	250 tokens
AUC-ROC	0.83	0.90	0.90	0.90	0.90
AUC-PR	0.34	0.50	0.56	0.56	0.57

As our study contains two classes of data that are imbalanced, AUC-ROC is not capable of fully reflecting model performance [29]. Fig 2(b) shows each model’s precision scores (the fraction of relevant articles among the retrieved articles) and recall scores (the fraction of the total amount of relevant articles that were actually retrieved) which better describe each model’s performance under data imbalance. The baseline model attained a precision of 0.268 and a recall of 0.645. This suggests that the linear Boolean search criterion did not well express the differences between the relevant and irrelevant documents. RF with refined clusters outperformed the baseline model at the same recall level and provided a precision of 0.268 and a recall of 0.731. Table 2 shows that RF with additional 20 important tokens improved AUC-PR from 0.34 to 0.50 compared to RF with refined clusters. Increasing the number of important tokens to 50 and 100 improved AUC-PR to 0.56, while increasing to 250 tokens achieved a peak of 0.57.

Under RF with top tokens, a recall of 0.8 can be achieved while maintaining a precision score of 0.4. This reduces the amount of manual reading for our example by 80%. Our example consists of about 10,000 total documents, about 1,000 of which are truly relevant, thus for RF with top tokens to correctly identify 800 relevant articles (80% of 1,000 truly relevant cases), 800/0.4 = 2,000 documents will be labeled as potentially relevant. Researchers can then manually read the 2,000 labeled documents to identify the 800 relevant articles, reducing the amount of manual reading from 10,000 to 2,000. Other cut-off probabilities could be explored and would lead to different combination precision and recall scores as illustrated in Fig 2(b). In practice, people could rank the probability of relevance, $\hat{P}\left(Y_{d}=1\right)$, and then prioritize manual reading of the highest probabilities. A stopping point for manual reading can be obtained through a heuristic, such as that used in SWIFT-Active Screening [30]. The above 5-fold cross-validation results reflect the model performance trained on 80% of the documents (10, 718 in total).

Finally, we compared random forest with other popular classifiers. As shown in Table 3, all with top 50 important tokens (features), random forest outperformed the SVM, Boosting (AdaBoost), Neural Network (multi-layer perceptron), and ElasticNet in terms of AUC-ROC and AUC-PR.

**Table 3:** The area under the curve (AUC) for ROC and PR of Random Forest, ElasticNet, Support Vector Machine (SVM), Neural Network and Boosting, all with top 50 tokens. They are presented in the decreasing order of AUC-PR.

Metric	Random Forest	ElasticNet	SVM	Neural Network	Boosting
AUC-ROC	0.90	0.89	0.88	0.85	0.86
AUC-PR	0.56	0.49	0.44	0.36	0.12

**Fig 3:**
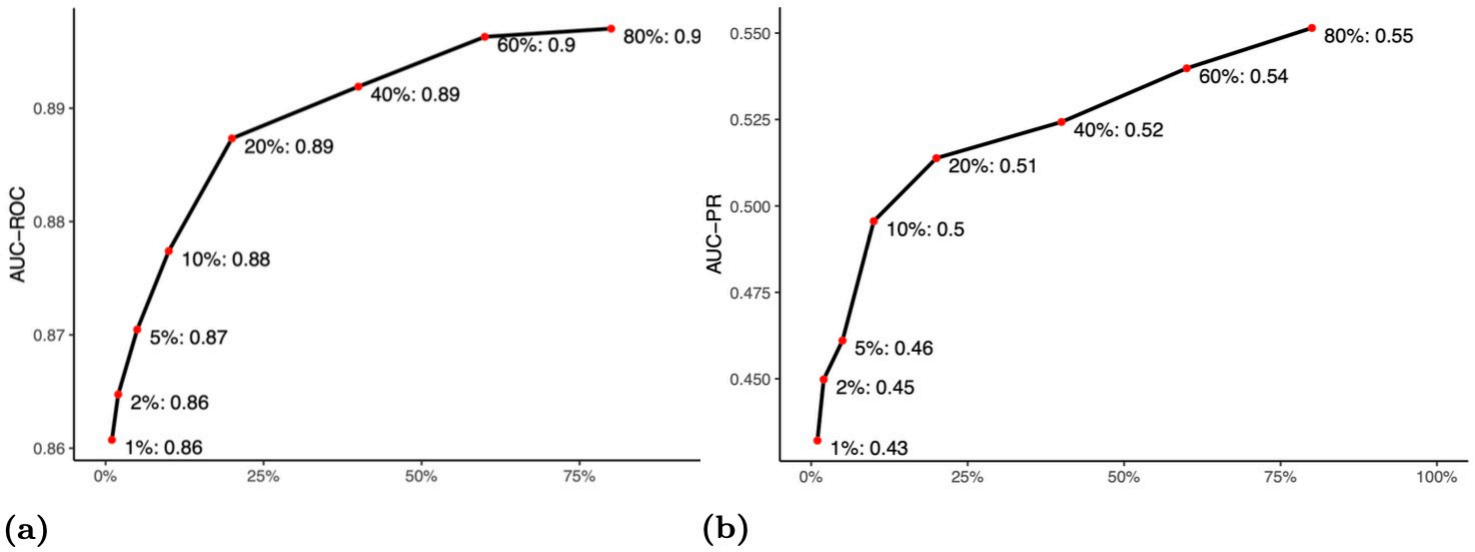
AUC for (a) ROC and (b) PR on testing data for RF with top 250 tokens with proportion of pre-labeled documents ranging from 1% to 80%.

Next, we investigated the training data sample size needed to achieve a good model performance. We use report AUC values for both ROC and PR curves. We trained RF with the top 250 significant tokens on different proportions of pre-labeled documents (10, 718 in total) from 1% up to 80% and used cross-validation to estimate the prediction accuracy on the test data. Fig 3 shows that we can attain an AUC for ROC above 80% using only 1% of pre-labeled documents (107 samples) as training data. The improvements for AUC-ROC and AUC-PR are marginal when 2, 144 (20% among 10, 718) or more pre-labeled documents were used.

It is useful to understand model performance from the perspective of the percentage of future work saved. The percentage of work saved oversampling at random is a metric commonly used to evaluate ADC models that screen documents for systematic reviews [31], often called WSS@*R*, where *R* is the desired recall level. If *P* is the total number of documents in the test data that were predicted relevant and *N* is the total number of test documents, then

$$\begin{array}{c} \text{WSS}@R=R-\frac{P}{N}. \end{array}$$ (2)

For RF with top tokens trained on 107 randomly selected training samples (1% of data), the average WSS@95 is 37%, and the average WSS@80 is 48%. With 1, 070 training samples (10% of data), the average WSS@95 is 42% and the average WSS@80 is 55%. As such, researchers can avoid reading 75% of future documents (68% of total) while retaining 80% of relevant documents.

## Discussion

Automatic document classification (ADC) can help distinguish relevant work from others, and many techniques have been developed and applied in this field, such as the Naïve Bayes classifier [32], support vector machines [33], and deep neural network methods [34]. As such, many papers in recent years have reviewed ADC techniques, discussing how they may be incorporated in the systematic review process [35–38]. The majority of ADC methods for screening papers in systematic reviews use the bag-of-words encoding of titles and abstracts, with a variety of classification algorithms of which support vector machine (SVM) [39, 40], Naïve Bayes, and ensemble methods [41] are the most widely-used [42]. We did not intend to provide a comprehensive comparison of ADC techniques, but instead we presented a case study where the feature screening and extraction can be extended to other classifiers and SR of other domain fields. We also illustrated where improvement of prediction accuracy came from by incrementally increasing the model complexity. New advances in ADC techniques would provide future improvement potentials for similar tasks.

By comparing the AUC from both ROC and PR curves, we found RF with top tokens is outperforming the other models. The sensitive analysis on training size also indicates that even with 10% of data being trained, RF with top tokens can still greatly reduce the human reading while keeping the recall rate as high as 80%.

The trained classifiers can also be used as a sanity test, as mistakes are inevitable when manually screening thousands of documents. Any disagreements between manual and automatic classification would be addressed with an additional layer of review. For instance, in some cases, our model assigned high probabilities of relevant/irrelevant to documents manually labeled as the opposite. We provided 40 such documents to the manual screening team for verification: 18 documents labeled as irrelevant and 22 documents labeled as relevant. Among the 18 marked irrelevant documents, five are indeed relevant but missed during the manual labeling process; among the 22 marked relevant documents, 15 of them are identified as “likely should not have been included”.

Note that we excluded papers that were not published in English; these results on using an automatic document classifier may not be generalizable to non-English language studies.

## Conclusions

The need to extract information through a systematic literature review arises in a wide range of domains. Here, we proposed multiple models for automatically identifying documents that characterize key features of female sex workers. Empirical results showed that using a random forest on semantic clusters of key tokens and an additional set of tokens outperformed others.

RF with top tokens could be applied to identifying other populations most affected by HIV, including clients of female sex workers, gay men and other men who have sex with men, people who use drugs, transgender populations, and incarcerated populations. We expect to obtain a reasonable classifier by taking 20% of retrieved documents as training samples. The proposed classifier could also be used in other research areas and for rapid documents screening on a tight schedule, such as COVID-related work during the crisis. We feel methods proposed here will help other researchers with such reviews making them faster to implement and more complete and enable reviews to readily scale to much larger numbers of documents.

## Supporting information

S1 Appendix (PDF)

S1 Checklist PRISMA 2020 checklist. (PDF)
