# Supplementary material for: Automating document classification with distant supervision to increase the efficiency of systematic reviews: A case study on identifying studies with HIV impacts on female sex workers
Source: PLoS One. 2022 Jun 30;17(6):e0270034. doi: 10.1371/journal.pone.0270034 (PMC9246134; doi:10.1371/journal.pone.0270034)
Supplement: S1 Appendix — (PDF) [file pone.0270034.s001.pdf]

PubMed

Concept 1: Female sex workers
"Prostitution"[MeSH] OR "Sex Workers"[MeSH] OR sex work*[tw] OR prostitut*[tw] OR "commercial sex"[tw] OR "transactional sex"[tw] OR "SW"[tw] OR "FSW"[tw] OR "CSW"[tw] OR "sex trade"[tw] OR "trade sex"[tw]

Concept 1: Men who have sex with men
"Homosexuality, Male"[Mesh] OR "gay men"[tw] OR "gay man"[tw] OR gay male*[tw] OR homosexual*[tw] OR "MSM"[tw] OR "men who have sex with men"[tw] OR "males who have sex with males"[tw] OR "bisexual men"[tw] OR "bisexual man"[tw] OR bisexual male*[tw]

Concept 1: People who use drugs
"Drug Users"[Mesh] OR "Substance Abuse, Intravenous"[Mesh] OR "drug use"[tw] OR "drug user"[tw] OR "drug users"[tw] OR drug abuse*[tw] OR substance use*[tw] OR substance abuse*[tw] OR people who inject drug*[tw] OR people who use drug*[tw] OR "IVDU"[tw] OR "IDU"[tw] OR "PWUD"[tw] OR "PWID"[tw] OR "drug usage"[tw]

Concept 1: Transgender people
"Transgender Persons"[Mesh] OR transgender*[tw] OR transsexual*[tw] OR transvest*[tw] OR travesti*[tw] OR cross dress*[tw] OR "koti"[tw] OR "hijra"[tw] OR "mahu"[tw] OR "waria"[tw] OR "katoey"[tw] OR "berdache"[tw] OR "male to female transgender"[tw] OR "MTF"[tw] OR transmascul*[tw]

Concept 1: Incarcerated populations
"Prisoners"[Mesh] OR incarcerat*[tw] OR prison*[tw]

Concept 2: HIV
"HIV"[Mesh] OR "Acquired Immunodeficiency Syndrome"[Mesh] OR "HIV Infections"[Mesh] OR human immunodeficiency virus*[tw] OR acquired immunodeficiency syndrome*[tw] OR HIV*[tw] OR "AIDS"[tw] OR HIV1*[tw] OR HIV2*[tw]

Concept 3: Violence
"Violence"[Mesh] OR "Domestic Violence"[Mesh] OR "Workplace Violence"[Mesh] OR "Crime Victims"[Mesh] OR "Battered Women"[Mesh] OR "Human Rights Abuses"[Mesh] OR "Sex Offenses"[Mesh] OR "Homicide"[Mesh] OR "Coercion"[Mesh] OR "Sexual Harassment"[Mesh] OR "Torture"[Mesh] OR "Spouse Abuse"[Mesh] OR "Rape"[Mesh] OR violen*[tw] OR crime*[tw] OR offense*[tw] OR abuse*[tw] OR victim*[tw] OR rape*[tw] OR assault*[tw] OR batter*[tw] OR extort*[tw] OR blackmail*[tw] OR intimidat*[tw] OR exploit*[tw] OR "IPV"[tw] OR "IPSV"[tw]

Date range
("2010/MM/DD"[PDAT] : "3000/MM/DD"[PDAT])

(CONCEPT 2 OR CONCEPT 3) AND CONCEPT 1=

Embase – DDMMYY

Concept 1: Female sex workers
'prostitution'/exp OR 'transactional sex'/exp OR ('sex work*' OR 'prostitut*' OR 'commercial sex' OR 'transactional sex' OR 'SW' OR 'FSW' OR 'CSW' OR 'sex trade' OR 'trade sex'):ab,ti

Concept 1: Men who have sex with men
'men who have sex with men'/exp OR 'homosexual male'/exp OR 'male homosexuality'/exp OR ('gay men' OR 'gay man' OR 'gay male*' OR 'homosexual*' OR 'MSM' OR 'men who have sex with men' OR 'males who have sex with males' OR 'bisexual men' OR 'bisexual man' OR 'bisexual male*'):ab,ti

Concept 1: People who use drugs
'intravenous drug abuse'/exp OR ('drug use' OR 'drug user' OR 'drug users' OR 'drug abuse*' OR 'substance use*' OR 'substance abuse*' OR 'people who inject drug*' OR 'people who use drug*' OR 'IVDU' OR 'IDU' OR 'PWUD' OR 'PWID' OR 'drug usage'):ab,ti

Concept 1: Transgender people
'transgender'/exp OR 'female to male transgender'/exp OR ('transgender*' OR 'transsexual*' OR 'transvest*' OR 'travesti*' OR 'cross dress*' OR 'koti' OR 'hijra' OR 'mahu' OR 'waria' OR 'katoey' OR 'berdache' OR 'male to female transgender' OR 'MTF' OR 'transmascul*'):ab,ti

Concept 1: Incarcerated populations
'prisoner'/exp OR ('incarcerat*' OR 'prison*'):ab,ti

Concept 2: HIV
'Human immunodeficiency virus'/exp OR 'acquired immune deficiency syndrome'/exp OR ('human immunodeficiency virus*' OR 'acquired immunodeficiency syndrome*' OR 'HIV*' OR 'AIDS' OR 'HIV1*' OR 'HIV2*'):ab,ti

Concept 3: Violence
'violence'/exp OR 'assault'/exp OR 'battering'/exp OR 'dating violence'/exp OR 'domestic violence'/exp OR 'partner violence'/exp OR 'gender based violence'/exp OR 'homicide'/exp OR 'physical violence'/exp OR 'sexual violence'/exp OR 'workplace violence'/exp OR 'torture'/exp OR ('violen*' OR 'crime*' OR 'offense*' OR 'abuse*' OR 'victim*' OR 'rape*' OR 'assault*' OR 'batter*' OR 'extort*' OR 'blackmail*' OR 'intimidat*' OR 'exploit*' OR 'IPV' OR 'IPSV'):ab,ti

DATE: (2006:py OR 2007:py OR 2008:py OR 2009:py OR 2010:py OR 2011:py OR 2012:py OR 2013:py OR 2014:py OR 2015:py OR 2016:py)

(CONCEPT 2 OR CONCEPT 3) AND CONCEPT 1=

Global Health – DDMMYY

Concept 1: Female sex workers
exp prostitution/ OR exp sex workers OR ("sex work*" OR "prostitut*" OR "commercial sex" OR "transactional sex" OR "SW" OR "FSW" OR "CSW" OR "sex trade" OR "trade sex").mp.

Concept 1: Men who have sex with men
exp men who have sex with men/ OR exp homosexuality/ OR ("gay men" OR "gay man" OR "gay male*" OR "homosexual*" OR "MSM" OR "men who have sex with men" OR "males who have sex with males" OR "bisexual men" OR "bisexual man" OR "bisexual male*").mp.

Concept 1: People who use drugs
exp drug users/ OR exp substance abuse/ OR ("drug use" OR "drug user" OR "drug users" OR "drug abuse*" OR "substance use*" OR "substance abuse*" OR "people who inject drug*" OR "people who use drug*" OR "IVDU" OR "IDU" OR "PWUD" OR "PWID" OR "drug usage").mp.

Concept 1: Transgender people
("transgender*" OR "transsexual*" OR "transvest*" OR "travesti*" OR "cross dress*" OR "koti" OR "hijra" OR "mahu" OR "waria" OR "katoey" OR "berdache" OR "male to female transgender" OR "MTF" OR "transmascul*").mp.

Concept 1: Incarcerated populations
exp prisoners/ OR (incarcerat* OR prison*).mp

Concept 2: HIV
exp human immunodeficiency viruses/ OR exp acquired immune deficiency syndrome/ OR ("human immunodeficiency virus*" OR "acquired immunodeficiency syndrome*" OR "HIV*" OR "AIDS" OR "HIV1*" OR "HIV2*").mp.

Concept 3: Violence

exp domestic violence/ OR exp rape/ OR exp sexual assault/ OR (violen* OR crime* OR offense* OR abuse* OR victim* OR rape* OR assault* OR batter* OR extort* OR blackmail* OR intimidat* OR exploit* OR "IPV" OR "IPSV").mp.

(CONCEPT 2 OR CONCEPT 3) AND CONCEPT 1=

SCOPUS – DDMMYY

Concept 1: Female sex workers
TITLE-ABS-KEY("sex work*" OR "prostitut*" OR "commercial sex" OR "transactional sex" OR "SW" OR "FSW" OR "CSW" OR "sex trade" OR "trade sex")

Concept 1: Men who have sex with men
TITLE-ABS-KEY("gay men" OR "gay man" OR "gay male*" OR "homosexual*" OR "MSM" OR "men who have sex with men" OR "males who have sex with males" OR "bisexual men" OR "bisexual man" OR "bisexual male*")

Concept 1: People who use drugs
TITLE-ABS-KEY("drug use" OR "drug user" OR "drug users" OR "drug abuse*" OR "substance use*" OR "substance abuse*" OR "people who inject drug*" OR "people who use drug*" OR "IVDU" OR "IDU" OR "PWUD" OR "PWID" OR "drug usage")

Concept 1: Transgender people
TITLE-ABS-KEY("transgender*" OR "transsexual*" OR "transvest*" OR "travesti*" OR "cross dress*" OR "koti" OR "hijra" OR "mahu" OR "waria" OR "katoey" OR "berdache" OR "male to female transgender" OR "MTF" OR "transmascul*")

Concept 1: Incarcerated populations
TITLE-ABS-KEY("incarcerat*" OR "prison*")

Concept 2: HIV
TITLE-ABS-KEY("human immunodeficiency virus*" OR "acquired immunodeficiency syndrome*" OR "HIV*" OR "AIDS" OR "HIV1*" OR "HIV2*")

Concept 3: Violence
TITLE-ABS-KEY("violen*" OR "crime*" OR "offense*" OR "abuse*" OR "victim*" OR "rape*" OR "assault*" OR "batter*" OR "extort*" OR "blackmail*" OR "intimidat*" OR "exploit*" OR "IPV" OR "IPSV")

PUBYEAR is 2006 OR PUBYEAR is 2007 OR PUBYEAR is 2008 OR PUBYEAR is 2009 OR PUBYEAR is 2010 OR PUBYEAR is 2011 OR PUBYEAR is 2012 OR PUBYEAR is 2013 OR PUBYEAR is 2014 OR PUBYEAR is 2015 OR PUBYEAR is 2016 OR PUBYEAR is 2017

(CONCEPT 2 OR CONCEPT 3) AND CONCEPT 1=

PsycINFO – DDMMYY

Concept 1: Female sex workers
DE "Prostitution" OR TI ("sex work*" OR "prostitut*" OR "commercial sex" OR "transactional sex" OR "SW" OR "FSW" OR "CSW" OR "sex trade" OR "trade sex") OR AB ("sex work*" OR "prostitut*" OR "commercial sex" OR "transactional sex" OR "SW" OR "FSW" OR "CSW" OR "sex trade" OR "trade sex") OR KW ("sex work*" OR "prostitut*" OR "commercial sex" OR "transactional sex" OR "SW" OR "FSW" OR "CSW" OR "sex trade" OR "trade sex")

Concept 1: Men who have sex with men
DE "Same Sex Intercourse" OR DE "Male Homosexuality" OR TI ("gay men" OR "gay man" OR "gay male*" OR "homosexual*" OR "MSM" OR "men who have sex with men" OR "males who have sex with males" OR "bisexual men" OR "bisexual man" OR "bisexual male*") OR AB ("gay men" OR "gay man" OR "gay male*" OR "homosexual*" OR "MSM" OR "men who have sex with men" OR "males who have sex with males" OR "bisexual men" OR "bisexual man" OR "bisexual male*") OR KW ("gay men" OR "gay man" OR "gay male*" OR "homosexual*" OR "MSM" OR "men who have sex with men" OR "males who have sex with males" OR "bisexual men" OR "bisexual man" OR "bisexual male*")

Concept 1: People who use drugs
DE "Drug Abuse" OR TI ("drug use" OR "drug user" OR "drug users" OR "drug abuse*" OR "substance use*" OR "substance abuse*" OR "people who inject drug*" OR "people who use drug*" OR "IVDU" OR "IDU" OR "PUD" OR "PWUD" OR "PWID" OR "drug usage") OR AB ("drug use" OR "drug user" OR "drug users" OR "drug abuse*" OR "substance use*" OR "substance abuse*" OR "people who inject drug*" OR "people who use drug*" OR "IVDU" OR "IDU" OR "PUD" OR "PWUD" OR "PWID" OR "drug usage") OR KW ("drug use" OR "drug user" OR "drug users" OR "drug abuse*" OR "substance use*" OR "substance abuse*" OR "people who inject drug*" OR "people who use drug*" OR "IVDU" OR "IDU" OR "PUD" OR "PWUD" OR "PWID" OR "drug usage")

Concept 1: Transgender people
DE "Transgender" OR TI ("transgender*" OR "transsexual*" OR "transvest*" OR "travesti*" OR "cross dress*" OR "koti" OR "hijra" OR "mahu" OR "waria" OR "katoey" OR "berdache" OR "male to female transgender" OR "MTF" OR "transmascul*") AB ("transgender*" OR "transsexual*" OR "transvest*" OR "travesti*" OR "cross dress*" OR "koti" OR "hijra" OR "mahu" OR "waria" OR "katoey" OR "berdache" OR "male to female transgender" OR "MTF" OR "transmascul*") KW ("transgender*" OR "transsexual*" OR "transvest*" OR "travesti*" OR "cross dress*" OR "koti" OR "hijra" OR "mahu" OR "waria" OR "katoey" OR "berdache" OR "male to female transgender" OR "MTF" OR "transmascul*")

Concept 1: Incarcerated populations
DE "Prisoners" OR TI ("incarcerat*" OR "prison*") OR AB ("incarcerat*" OR "prison*") OR KW ("incarcerat*" OR "prison*")

Concept 2: HIV
DE "HIV" OR DE "AIDS" OR TI ("human immunodeficiency virus*" OR "acquired immunodeficiency syndrome*" OR "HIV*" OR "AIDS" "HIV1*" OR "HIV2*") OR AB ("human immunodeficiency virus*" OR "acquired immunodeficiency syndrome*" OR "HIV*" OR "AIDS" "HIV1*" OR "HIV2*") OR KW ("human immunodeficiency virus*" OR "acquired immunodeficiency syndrome*" OR "HIV*" OR "AIDS" "HIV1*" OR "HIV2*")

Concept 3: Violence

DE "Violence" OR DE "Domestic Violence" OR DE "Intimate Partner Violence" OR DE "Violent Crime" OR DE "Workplace Violence" OR DE "Rape" OR DE "Acquaintance Rape" OR DE "Sex Offenses" OR TI ("violen*" OR "crime*" OR "offense*" OR "abuse*" OR "victim*" OR "rape*" OR "assault*" OR "batter*" OR "extort*" OR "blackmail*" OR "intimidat*" OR "exploit*" OR "IPV" OR "IPSV") OR AB ("violen*" OR "crime*" OR "offense*" OR "abuse*" OR "victim*" OR "rape*" OR "assault*" OR "batter*" OR "extort*" OR "blackmail*" OR "intimidat*" OR "exploit*" OR "IPV" OR "IPSV") OR KW ("violen*" OR "crime*" OR "offense*" OR "abuse*" OR "victim*" OR "rape*" OR "assault*" OR "batter*" OR "extort*" OR "blackmail*" OR "intimidat*" OR "exploit*" OR "IPV" OR "IPSV")

(CONCEPT 2 OR CONCEPT 3) AND CONCEPT 1=

Sociological Abstracts – DDMMYY

Concept 1: Female sex workers

SU.EXACT("Prostitution") OR xm,&wi|${ svo.&$S V$&tvswxmxyx.&$S V$&gsq q ivgmep$wi|&$S V$ &xerwegxmsrep$wi|&$S V$&W[&$S V$&JW[&$S V$&GW[&$S V$&wi|$xehi&$S V$&xehi$wi|&-$S V$ef,&wi|$ { svo.&$S V$&tvswxmxyx.&$S V$&gsq q ivgmep$wi|&$S V$&xerwegxmsrep$wi|&$S V$&W[&$S V$&JW[&$S V$ &GW[&$S V$&wi|$xehi&$S V$&xehi$wi|&-$S V$wy,&wi|${ svo.&$S V$&tvswxmxyx.&$S V$&gsq q ivgmep$wi|&$ S V$&xerwegxmsrep$wi|&$S V$&W[&$S V$&JW[&$S V$&GW[&$S V$&wi|$xehi&$S V$&xehi$wi|&-

Concept 1: Men who have sex with men

SU.EXACT("Homosexuality") OR ti("gay men" OR "gay man" OR "gay male*" OR "homosexual*" OR "MSM" OR "men who have sex with men" OR "males who have sex with males" OR "bisexual men" OR "bisexual man" OR "bisexual male*") OR ab("gay men" OR "gay man" OR "gay male*" OR "homosexual*" OR "MSM" OR "men who have sex with men" OR "males who have sex with males" OR "bisexual men" OR "bisexual man" OR "bisexual male*") OR su("gay men" OR "gay man" OR "gay male*" OR "homosexual*" OR "MSM" OR "men who have sex with men" OR "males who have sex with males" OR "bisexual men" OR "bisexual man" OR "bisexual male*")

Concept 1: People who use drugs

SU.EXACT("Drug Abuse") OR ti("drug use" OR "drug user" OR "drug users" OR "drug abuse*" OR "substance use*" OR "substance abuse*" OR "people who inject drug*" OR "people who use drug*" OR "IVDU" OR "IDU" OR "PUD" OR "PWUD" OR "PWID" OR "drug usage") OR ab("drug use" OR "drug user" OR "drug users" OR "drug abuse*" OR "substance use*" OR "substance abuse*" OR "people who inject drug*" OR "people who use drug*" OR "IVDU" OR "IDU" OR "PUD" OR "PWUD" OR "PWID" OR "drug usage") OR su("drug use" OR "drug user" OR "drug users" OR "drug abuse*" OR "substance use*" OR "substance abuse*" OR "people who inject drug*" OR "people who use drug*" OR "IVDU" OR "IDU" OR "PUD" OR "PWUD" OR "PWID" OR "drug usage")

Concept 1: Transgender people

SU.EXACT("Transvestism") OR ti("transgender*" OR "transsexual*" OR "transvest*" OR "travesti*" OR "cross dress*" OR "koti" OR "hijra" OR "mahu" OR "waria" OR "katoey" OR "berdache" OR "male to female transgender" OR "MTF" OR "transmascul*") OR ab("transgender*" OR "transsexual*" OR "transvest*" OR "travesti*" OR "cross dress*" OR "koti" OR "hijra" OR "mahu" OR "waria" OR "katoey" OR "berdache" OR "male to female transgender" OR "MTF" OR "transmascul*") OR su("transgender*" OR "transsexual*" OR "transvest*" OR "travesti*" OR "cross dress*" OR "koti" OR "hijra" OR "mahu" OR "waria" OR "katoey" OR "berdache" OR "male to female transgender" OR "MTF" OR "transmascul*")

Concept 1: Incarcerated populations

SU.EXACT("Prisoners") OR ti("incarcerat*" OR "prison*") OR ab("incarcerat*" OR "prison*") OR su("incarcerat*" OR "prison*")

Concept 2: HIV

SU.EXACT("Acquired Immune Deficiency Syndrome") OR ti("human immunodeficiency virus*" OR "acquired immunodeficiency syndrome*" OR "HIV*" OR "AIDS" OR "HIV1*" OR "HIV2*") OR ab("human immunodeficiency virus*" OR "acquired immunodeficiency syndrome*" OR "HIV*" OR "AIDS" OR "HIV1*" OR "HIV2*") OR su("human immunodeficiency virus*" OR "acquired immunodeficiency syndrome*" OR "HIV*" OR "AIDS" OR "HIV1*" OR "HIV2*")

Concept 3: Violence

SU.EXACT("Sexual Assault") OR SU.EXACT("Violence") OR SU.EXACT("Family Violence") OR ti("violen*" OR "crime*" OR "offense*" OR "abuse*" OR "victim*" OR "rape*" OR "assault*" OR "batter*" OR "extort*" OR "blackmail*" OR "intimidat*" OR "exploit*" OR "IPV" OR "IPSV") OR ab("violen*" OR "crime*" OR "offense*" OR "abuse*" OR "victim*" OR "rape*" OR "assault*" OR "batter*" OR "extort*" OR "blackmail*" OR "intimidat*" OR "exploit*" OR "IPV" OR "IPSV") OR su("violen*" OR "crime*" OR "offense*" OR "abuse*" OR "victim*" OR "rape*" OR "assault*" OR "batter*" OR "extort*" OR "blackmail*" OR "intimidat*" OR "exploit*" OR "IPV" OR "IPSV")

(CONCEPT 2 OR CONCEPT 3) AND CONCEPT 1=

CINAHL – DDMMYY

Concept 1: Female sex workers
MH "Prostitution" OR TI ("sex work*" OR "prostitut*" OR "commercial sex" OR "transactional sex" OR "SW" OR "FSW" OR "CSW" OR "sex trade" OR "trade sex") OR AB ("sex work*" OR "prostitut*" OR "commercial sex" OR "transactional sex" OR "SW" OR "FSW" OR "CSW" OR "sex trade" OR "trade sex") OR SU ("sex work*" OR "prostitut*" OR "commercial sex" OR "transactional sex" OR "SW" OR "FSW" OR "CSW" OR "sex trade" OR "trade sex")

Concept 1: Men who have sex with men
MH "Homosexuals, Male" OR TI ("gay men" OR "gay man" OR "gay male*" OR "homosexual*" OR "MSM" OR "men who have sex with men" OR "males who have sex with males" OR "bisexual men" OR "bisexual man" OR "bisexual male*") OR AB ("gay men" OR "gay man" OR "gay male*" OR "homosexual*" OR "MSM" OR "men who have sex with men" OR "males who have sex with males" OR "bisexual men" OR "bisexual man" OR "bisexual male*") OR KW ("gay men" OR "gay man" OR "gay male*" OR "homosexual*" OR "MSM" OR "men who have sex with men" OR "males who have sex with males" OR "bisexual men" OR "bisexual man" OR "bisexual male*")

Concept 1: People who use drugs
MH "Substance Abuse" OR TI ("drug use" OR "drug user" OR "drug users" OR "drug abuse*" OR "substance use*" OR "substance abuse*" OR "people who inject drug*" OR "people who use drug*" OR "IVDU" OR "IDU" OR "PUD" OR "PWUD" OR "PWID" OR "drug usage") OR AB ("drug use" OR "drug user" OR "drug users" OR "drug abuse*" OR "substance use*" OR "substance abuse*" OR "people who inject drug*" OR "people who use drug*" OR "IVDU" OR "IDU" OR "PUD" OR "PWUD" OR "PWID" OR "drug usage") OR KW ("drug use" OR "drug user" OR "drug users" OR "drug abuse*" OR "substance use*" OR "substance abuse*" OR "people who inject drug*" OR "people who use drug*" OR "IVDU" OR "IDU" OR "PUD" OR "PWUD" OR "PWID" OR "drug usage")

Concept 1: Transgender people
MH "Transgendered persons" OR TI ("transgender*" OR "transsexual*" OR "transvest*" OR "travesti*" OR "cross dress*" OR "koti" OR "hijra" OR "mahu" OR "waria" OR "katoey" OR "berdache" OR "male to female transgender" OR "MTF" OR "transmascul*") AB ("transgender*" OR "transsexual*" OR "transvest*" OR "travesti*" OR "cross dress*" OR "koti" OR "hijra" OR "mahu" OR "waria" OR "katoey" OR "berdache" OR "male to female transgender" OR "MTF" OR "transmascul*") KW ("transgender*" OR "transsexual*" OR "transvest*" OR "travesti*" OR "cross dress*" OR "koti" OR "hijra" OR "mahu" OR "waria" OR "katoey" OR "berdache" OR "male to female transgender" OR "MTF" OR "transmascul*")

Concept 1: Incarcerated populations
MH "Prisoners" OR TI ("incarcerat*" OR "prison*") OR AB ("incarcerat*" OR "prison*") OR KW ("incarcerat*" OR "prison*")

Concept 2: HIV
MH "Human Immunodeficiency Virus" OR MH "Acquired Immunodeficiency Syndrome" OR TI ("human immunodeficiency virus*" OR "acquired immunodeficiency syndrome*" OR "HIV*" OR "AIDS" "HIV1*" OR "HIV2*") OR AB ("human immunodeficiency virus*" OR "acquired immunodeficiency syndrome*" OR "HIV*" OR "AIDS" "HIV1*" OR "HIV2*") OR KW ("human immunodeficiency virus*" OR "acquired immunodeficiency syndrome*" OR "HIV*" OR "AIDS" "HIV1*" OR "HIV2*")

Concept 3: Violence

MH "Violence" OR MH "Workplace Violence" OR MH "Domestic Violence" OR MH "Intimate Partner Violence" OR MH "Rape" OR TI ("violen*" OR "crime*" OR "offense*" OR "abuse*" OR "victim*" OR "rape*" OR "assault*" OR "batter*" OR "extort*" OR "blackmail*" OR "intimidat*" OR "exploit*" OR "IPV" OR "IPSV") OR AB ("violen*" OR "crime*" OR "offense*" OR "abuse*" OR "victim*" OR "rape*" OR "assault*" OR "batter*" OR "extort*" OR "blackmail*" OR "intimidat*" OR "exploit*" OR "IPV" OR "IPSV") OR KW ("violen*" OR "crime*" OR "offense*" OR "abuse*" OR "victim*" OR "rape*" OR "assault*" OR "batter*" OR "extort*" OR "blackmail*" OR "intimidat*" OR "exploit*" OR "IPV" OR "IPSV")

(CONCEPT 2 OR CONCEPT 3) AND CONCEPT 1=

Web of Science – DDMMYY

Concept 1: Female sex workers
TS=("sex work*" OR "prostitut*" OR "commercial sex" OR "transactional sex" OR "SW" OR "FSW" OR "CSW" OR "sex trade" OR "trade sex")

Concept 1: Men who have sex with men
TS=("gay men" OR "gay man" OR "gay male*" OR "homosexual*" OR "MSM" OR "men who have sex with men" OR "males who have sex with males" OR "bisexual men" OR "bisexual man" OR "bisexual male*")

Concept 1: People who use drugs
TS=("drug use" OR "drug user" OR "drug users" OR "drug abuse*" OR "substance use*" OR "substance abuse*" OR "people who inject drug*" OR "people who use drug*" OR "IVDU" OR "IDU" OR "PUD" OR "PWUD" OR "PWID" OR "drug usage")

Concept 1: Transgender people
TS=("transgender*" OR "transsexual*" OR "transvest*" OR "travesti*" OR "cross dress*" OR "koti" OR "hijra" OR "mahu" OR "waria" OR "katoey" OR "berdache" OR "male to female transgender" OR "MTF" OR "transmascul*")

Concept 1: Incarcerated populations
TS=("incarcerat*" OR "prison*")

Concept 2: HIV
TS=("human immunodeficiency virus*" OR "acquired immunodeficiency syndrome*" OR "HIV*" OR "AIDS" OR "HIV1*" OR "HIV2*")

Concept 3: Violence
TS=("violen*" OR "crime*" OR "offense*" OR "abuse*" OR "victim*" OR "rape*" OR "assault*" OR "batter*" OR "extort*" OR "blackmail*" OR "intimidat*" OR "exploit*" OR "IPV" OR "IPSV")

Date Limit:
PY=2006-2016 (see if you can leave off the 2016)

(CONCEPT 2 OR CONCEPT 3) AND CONCEPT 1=

POPLine – DDMMYY (no need for field tags)

Concept 1: Female sex workers
"Sex workers" OR "sex work*" OR "prostitut*" OR "commercial sex" OR "transactional sex" OR "SW" OR "FSW" OR "CSW" OR "sex trade" OR "trade sex"

Concept 1: Men who have sex with men
"Men having sex with men" OR "homosexuals" OR "gay men" OR "gay man" OR "gay male*" OR "homosexual*" OR "MSM" OR "men who have sex with men" OR "males who have sex with males" OR "bisexual men" OR "bisexual man" OR "bisexual male*"

Concept 1: People who use drugs
"Drug Use and Abuse" OR "IV drug users" OR "drug use*" OR "drug abuse*" OR "substance use*" OR "substance abuse*" OR "people who inject drug*" OR "people who use drug*" OR "IVDU" OR "IDU" OR "PUD" OR "PWUD" OR "PWID" OR "drug usage"

Concept 1: Transgender people
"transgender*" OR "transsexual*" OR "transvest*" OR "travesti*" OR "cross dress*" OR "koti" OR "hijra" OR "mahu" OR "waria" OR "katoey" OR "berdache" OR "male to female transgender" OR "MTF" OR "transmascul*"

Concept 1: Incarcerated populations
"prisoners" OR "incarcerat*" OR "prison*"

Concept 2: HIV
"HIV" OR "human immunodeficiency virus*" OR "acquired immunodeficiency syndrome*" OR "HIV*" OR "AIDS" OR "HIV1*" OR "HIV2*"

Concept 3: Violence
"Violence" OR "Domestic Violence" OR "Rape" OR "Homicide" OR "Sexual Abuse" OR "Sexual Harassment" OR "violen*" OR "crime*" OR "offense*" OR "abuse*" OR "victim*" OR "rape*" OR "assault*" OR "batter*" OR "extort*" OR "blackmail*" OR "intimidat*" OR "exploit*" OR "IPV" OR "IPSV"

(CONCEPT 2 OR CONCEPT 3) AND CONCEPT 1=
